# College and Hospital Notes

**Published:** 1906-07

**Authors:** 


### COLLEGE AND HOSPITAL NOTES.

The Buffalo Fresh Air Mission Hospital at Athol Springs will open July 1, 1906. The hospital is particularly designed for choleraic infants, but in view of the remarkable results obtained from fresh air treatment of children with tuberculosis of the glands and bones, especially in France and Germany, and the recently established hospital at Sea Breeze, near New York, the hospital staff has decided to use the Fresh Air Mission Hospital this summer very largely for the care of such children. Children up to 15 years of age will be received. Application should be made at the office of the Fresh Air Mission, 19 West Tupper Street, Buffalo, where arrangements will be made for the examination of the children desiring admission. No charge will be made for transportation to and from the hospital, or for treatment given.

At the Buffalo Hospital of the Sisters of Charity, the following named physicians have passed examination for the position of internes: J. H. Donnely, Buffalo, (McGill Univ.) ; Joseph Brady, Boston, (Yale Univ.) ; John Smith, Ann Arbor, (Univ. Mich.) ; Harvey W. Bodamer, John C. Hoeffler, and Winfield A. Peterson (Univ. Buffalo).

The Buffalo General Hospital Training School for Nurses held commencement exercises on the evening of Tuesday, June 12, 1906, at which twenty-one young women received diplomas. The ceremonies took place in the gymnasium of the nurses' home on High street and were interspersed with music. Mr. Charles W. Pardee, president of the hospital board, awarded the diplomas, Professor F. Hyatt Smith made an address, and Dr. William C. Krauss presented the badges.

The Buffalo Homeopathic Hospital Training School for Nurses held commencement exercises at the First Presbyterian Church chapel on the evening of June 11, 1906, at which eight young women received diplomas. Dr. George R. Critchlow presented the class, diplomas were awarded by Martin Clark, secretary of the trustees, badges were presented by Dr. Burt J. Maycock, and Dr. F. C. Busch supplied vocal music. After the exercises were over a reception was held at the chapter house in Johnson Park, refreshments were served and music and dancing from 9:30 to 11 p. m. closed an interesting evening.

